# *N*-Benzyl-*N*-cyclo­hexyl-4-methyl­benzene­sulfonamide

**DOI:** 10.1107/S1600536809048193

**Published:** 2009-11-18

**Authors:** Islam Ullah Khan, Zeeshan Haider, Muhammad Zia-ur-Rehman, Muhammad Shafiq, Muhammad Nadeem Arshad

**Affiliations:** a Department of Chemistry, Government College University, Lahore 54000, Pakistan; b Applied Chemistry Research Centre, PCSIR Laboratories Complex, Ferozpure Road, Lahore 54600, Pakistan

## Abstract

In the title compound, C_20_H_25_NO_2_S, the cyclo­hexyl ring exists in a chair form and the mean plane through all six atoms makes dihedral angles of 56.12 (9) and 55.19 (10)° with the benzene and phenyl rings, respectively. The dihedral angle between the two aromatic rings is 77.23 (7)°. A weak intra­molecular C—H⋯O interaction occurs.

## Related literature

For the biological activity of sulfonamides, see: Ozbek *et al.* (2007 ▶); Parari *et al.* (2008 ▶); Ratish *et al.* (2009 ▶); Selnam *et al.* (2001 ▶). For related structures, see: Khan *et al.* (2009 ▶); Zia-ur-Rehman *et al.* (2009 ▶); Gowda *et al.* (2007*a* ▶,*b* ▶,*c* ▶). For bond-length data, see: Allen *et al.* (1987 ▶).
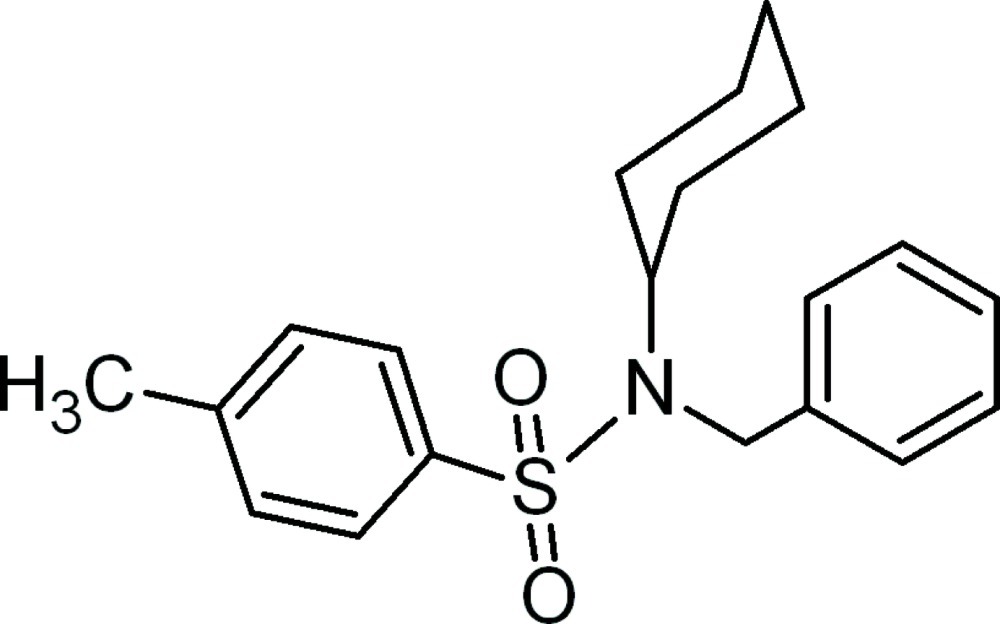


## Experimental

### 

#### Crystal data

C_20_H_25_NO_2_S
*M* *_r_* = 343.47
Orthorhombic, 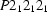

*a* = 9.0702 (4) Å
*b* = 11.1054 (5) Å
*c* = 18.1971 (8) Å
*V* = 1832.96 (14) Å^3^
*Z* = 4
Mo *K*α radiation
μ = 0.19 mm^−1^
*T* = 296 K
0.24 × 0.18 × 0.13 mm

#### Data collection

Bruker APEXII CCD area-detector diffractometer
Absorption correction: multi-scan (**SADABS**; Sheldrick, 1996 ▶) *T*_min_ = 0.956, *T*_max_ = 0.976
11619 measured reflections
4493 independent reflections
2764 reflections with *I* > 2σ(*I*)
*R*_int_ = 0.036

#### Refinement

*R*[*F* ^2^ > 2σ(*F* ^2^)] = 0.049
*wR*(*F* ^2^) = 0.097
*S* = 0.98
4493 reflections
218 parameters
H-atom parameters constrained
Δρ_max_ = 0.16 e Å^−3^
Δρ_min_ = −0.25 e Å^−3^
Absolute structure: Flack (1983 ▶), 1915 Friedel pairs
Flack parameter: 0.04 (8)

### 

Data collection: *APEX2* (Bruker, 2007 ▶); cell refinement: *SAINT* (Bruker, 2007 ▶); data reduction: *SAINT*; program(s) used to solve structure: *SHELXS97* (Sheldrick, 2008 ▶); program(s) used to refine structure: *SHELXL97* (Sheldrick, 2008 ▶); molecular graphics: *PLATON* (Spek, 2009 ▶) and *Mercury* (Macrae *et al.*, 2006 ▶); software used to prepare material for publication: *WinGX* (Farrugia, 1999 ▶) and *PLATON*.

## Supplementary Material

Crystal structure: contains datablocks I, New_Global_Publ_Block. DOI: 10.1107/S1600536809048193/is2488sup1.cif

Structure factors: contains datablocks I. DOI: 10.1107/S1600536809048193/is2488Isup2.hkl

Additional supplementary materials:  crystallographic information; 3D view; checkCIF report

## Figures and Tables

**Table 1:** Hydrogen-bond geometry (Å, °)

*D*—H⋯*A*	*D*—H	H⋯*A*	*D*⋯*A*	*D*—H⋯*A*
C7—H7⋯O1	0.98	2.38	2.903 (3)	113

**Fig. 1.**
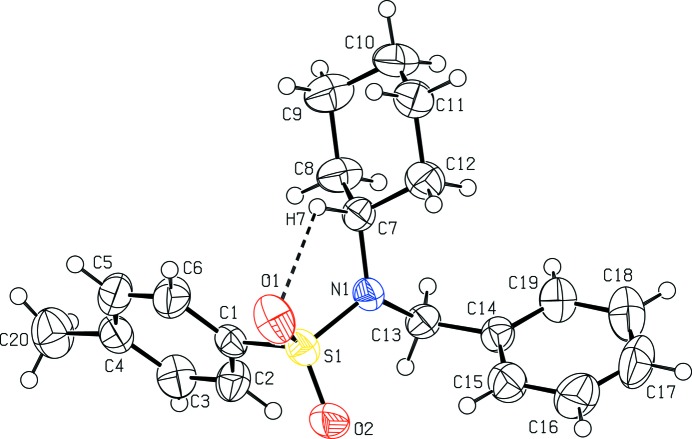
The molecular structure of (I), with displacement ellipsoids at the 50% probability level.

## supplementary crystallographic information

### Comment

Sulfonamides are well known as anti-inflamatory (Ratish *et al.*, 2009), anti-microbial (Ozbek *et al.*, 2007; Parari *et al.*, 2008), anti HIV (Selnam *et al.*, 2001) compounds. In continuation of our work regarding the synthesis of various sulfur containing heterocycles (Zia-ur-Rehman *et al.*, 2009; Khan *et al.*, 2009), the structure of *N*-benzyl-*N*-cyclohexyl-4-methyl benzene sulfonamide, (I), has been determined.

Bond lengths and bond angles of the title molecule (Fig. 1) are almost similar to those in the related molecules (Gowda *et al.*, 2007*a*,*b*,*c*) and are within the normal ranges (Allen *et al.*, 1987). The two aromatic rings as usual are essentially planar, while the cyclohexane ring is in a chair form. The dihedral angles between the two aromatic rings (C1—C6) & (C14—C19), the benzene (C1—C6) ring & the mean plane of cyclohexyl ring (C7—C12), and the phenyl (C14—C19) ring & the mean plane cyclohexyl ring (C7—C12) are 77.23 (7), 56.12 (9) and 55.19 (10)°, respectively, while the r.m.s. deviations for the (C1—C6), (C7—C12) & (C14—C19) rings are 0.0056, 0.2320 and 0.0046 Å, respectively. An intramolecular C—H···O hydrogen bond gives rise to a five membered hydrogen bonded ring (Table 1).

### Experimental

A mixture of *N*-cyclohexyl-4-methyl benzene sulfonamide (1.089 g, 4.3 mmol), sodium hydride (0.21 g, 0.88 mmol) and *N*, *N*-dimethylformamide (10 ml) was stirred at room temperature for half an hour followed by addition of benzyl chloride (1.14 g, 9.0 mmol). Stirring was continued further for a period of three hours and the contents were poured over crushed ice. Precipitated product was isolated, washed and crystallized from a methanol solution.

### Refinement

All H atoms were identified in a difference map and then were treated as riding (C—H = 0.93–0.98 Å), with *U*_iso_(H) = 1.2*U*_eq_(C) or 1.5*U*_eq_(methyl C).

### Crystal data

**Table:**

C_20_H_25_NO_2_S	*F*(000) = 736
*M**_r_* = 343.47	*D*_x_ = 1.245 Mg m^−^^3^
Orthorhombic, *P*2_1_2_1_2_1_	Mo *K*α radiation, λ = 0.71073 Å
Hall symbol: P 2ac 2ab	Cell parameters from 2246 reflections
*a* = 9.0702 (4) Å	θ = 2.9–20.7°
*b* = 11.1054 (5) Å	µ = 0.19 mm^−^^1^
*c* = 18.1971 (8) Å	*T* = 296 K
*V* = 1832.96 (14) Å^3^	Blocks, yellow
*Z* = 4	0.24 × 0.18 × 0.13 mm

### Data collection

**Table:**

Bruker APEXII CCD area-detector diffractometer	4493 independent reflections
Radiation source: fine-focus sealed tube	2764 reflections with *I* > 2σ(*I*)
graphite	*R*_int_ = 0.036
φ and ω scans	θ_max_ = 28.3°, θ_min_ = 2.9°
Absorption correction: multi-scan (*SADABS*; Sheldrick, 1996)	*h* = −12→12
*T*_min_ = 0.956, *T*_max_ = 0.976	*k* = −14→7
11619 measured reflections	*l* = −24→22

### Refinement

**Table:**

Refinement on *F*^2^	Secondary atom site location: difference Fourier map
Least-squares matrix: full	Hydrogen site location: inferred from neighbouring sites
*R*[*F*^2^ > 2σ(*F*^2^)] = 0.049	H-atom parameters constrained
*wR*(*F*^2^) = 0.097	*w* = 1/[σ^2^(*F*_o_^2^) + (0.0397*P*)^2^] where *P* = (*F*_o_^2^ + 2*F*_c_^2^)/3
*S* = 0.98	(Δ/σ)_max_ < 0.001
4493 reflections	Δρ_max_ = 0.16 e Å^−^^3^
218 parameters	Δρ_min_ = −0.24 e Å^−^^3^
0 restraints	Absolute structure: Flack (1983), 1915 Friedel pairs
Primary atom site location: structure-invariant direct methods	Flack parameter: 0.04 (8)

### Special details

**Table:**

Geometry. All e.s.d.'s (except the e.s.d. in the dihedral angle between two l.s. planes) are estimated using the full covariance matrix. The cell e.s.d.'s are taken into account individually in the estimation of e.s.d.'s in distances, angles and torsion angles; correlations between e.s.d.'s in cell parameters are only used when they are defined by crystal symmetry. An approximate (isotropic) treatment of cell e.s.d.'s is used for estimating e.s.d.'s involving l.s. planes.
Refinement. Refinement of *F*^2^ against ALL reflections. The weighted *R*-factor *wR* and goodness of fit *S* are based on *F*^2^, conventional *R*-factors *R* are based on *F*, with *F* set to zero for negative *F*^2^. The threshold expression of *F*^2^ > σ(*F*^2^) is used only for calculating *R*-factors(gt) *etc*. and is not relevant to the choice of reflections for refinement. *R*-factors based on *F*^2^ are statistically about twice as large as those based on *F*, and *R*- factors based on ALL data will be even larger.

### Fractional atomic coordinates and isotropic or equivalent isotropic displacement parameters (Å^2^)

**Table:**

	*x*	*y*	*z*	*U*_iso_*/*U*_eq_	
S1	0.13668 (7)	0.35270 (6)	0.83512 (3)	0.04431 (17)	
O1	0.0611 (2)	0.45762 (15)	0.86060 (9)	0.0603 (5)	
O2	0.28860 (18)	0.36124 (17)	0.81433 (9)	0.0604 (5)	
N1	0.04730 (19)	0.30283 (17)	0.76418 (9)	0.0396 (5)	
C1	0.1256 (3)	0.2456 (2)	0.90607 (11)	0.0386 (5)	
C2	0.2185 (3)	0.1481 (2)	0.90729 (13)	0.0520 (6)	
H2	0.2872	0.1375	0.8699	0.062*	
C3	0.2102 (3)	0.0660 (2)	0.96364 (14)	0.0556 (7)	
H3	0.2729	−0.0002	0.9632	0.067*	
C4	0.1123 (3)	0.0789 (2)	1.02044 (13)	0.0492 (6)	
C5	0.0189 (3)	0.1763 (3)	1.01803 (13)	0.0634 (8)	
H5	−0.0498	0.1867	1.0554	0.076*	
C6	0.0247 (3)	0.2591 (3)	0.96159 (13)	0.0607 (8)	
H6	−0.0399	0.3241	0.9612	0.073*	
C7	−0.1161 (2)	0.3031 (2)	0.76700 (11)	0.0404 (6)	
H7	−0.1444	0.3559	0.8078	0.049*	
C8	−0.1825 (2)	0.1808 (2)	0.78275 (15)	0.0573 (7)	
H8A	−0.1451	0.1507	0.8292	0.069*	
H8B	−0.1539	0.1246	0.7445	0.069*	
C9	−0.3505 (3)	0.1892 (3)	0.78615 (16)	0.0700 (8)	
H9A	−0.3916	0.1094	0.7933	0.084*	
H9B	−0.3790	0.2385	0.8278	0.084*	
C10	−0.4124 (3)	0.2434 (3)	0.71630 (16)	0.0723 (9)	
H10A	−0.3913	0.1904	0.6752	0.087*	
H10B	−0.5186	0.2508	0.7207	0.087*	
C11	−0.3468 (3)	0.3647 (3)	0.70192 (14)	0.0630 (8)	
H11A	−0.3752	0.4195	0.7409	0.076*	
H11B	−0.3852	0.3961	0.6560	0.076*	
C12	−0.1796 (2)	0.3582 (3)	0.69771 (13)	0.0556 (7)	
H12A	−0.1509	0.3100	0.6556	0.067*	
H12B	−0.1398	0.4385	0.6911	0.067*	
C13	0.1222 (3)	0.2229 (2)	0.71193 (11)	0.0423 (6)	
H13A	0.0613	0.1522	0.7043	0.051*	
H13B	0.2143	0.1964	0.7336	0.051*	
C14	0.1544 (2)	0.2793 (2)	0.63832 (12)	0.0412 (6)	
C15	0.2396 (3)	0.3820 (2)	0.63277 (14)	0.0582 (8)	
H15	0.2745	0.4194	0.6751	0.070*	
C16	0.2731 (3)	0.4293 (3)	0.56453 (18)	0.0753 (9)	
H16	0.3311	0.4981	0.5614	0.090*	
C17	0.2222 (4)	0.3763 (3)	0.50164 (17)	0.0773 (10)	
H17	0.2457	0.4082	0.4559	0.093*	
C18	0.1365 (4)	0.2759 (3)	0.50727 (15)	0.0756 (9)	
H18	0.1007	0.2393	0.4649	0.091*	
C19	0.1022 (3)	0.2279 (2)	0.57493 (14)	0.0572 (7)	
H19	0.0430	0.1597	0.5777	0.069*	
C20	0.1098 (3)	−0.0076 (3)	1.08385 (14)	0.0736 (9)	
H20A	0.0270	0.0100	1.1149	0.110*	
H20B	0.1993	0.0003	1.1116	0.110*	
H20C	0.1015	−0.0884	1.0656	0.110*	

### Atomic displacement parameters (Å^2^)

**Table:**

	*U*^11^	*U*^22^	*U*^33^	*U*^12^	*U*^13^	*U*^23^
S1	0.0498 (4)	0.0418 (3)	0.0414 (3)	−0.0050 (3)	−0.0074 (3)	−0.0030 (3)
O1	0.0852 (13)	0.0385 (10)	0.0573 (12)	0.0057 (9)	−0.0130 (9)	−0.0102 (9)
O2	0.0487 (10)	0.0769 (14)	0.0555 (11)	−0.0207 (10)	−0.0072 (8)	0.0060 (10)
N1	0.0385 (11)	0.0467 (13)	0.0337 (11)	0.0014 (9)	−0.0024 (8)	−0.0059 (9)
C1	0.0404 (13)	0.0399 (14)	0.0353 (12)	−0.0011 (12)	−0.0071 (11)	−0.0064 (10)
C2	0.0575 (15)	0.0552 (16)	0.0432 (15)	0.0053 (15)	0.0086 (11)	−0.0058 (15)
C3	0.0648 (17)	0.0481 (17)	0.0538 (17)	0.0098 (14)	−0.0022 (14)	−0.0005 (14)
C4	0.0535 (17)	0.0530 (17)	0.0413 (14)	−0.0061 (14)	−0.0065 (13)	0.0013 (12)
C5	0.0583 (17)	0.089 (3)	0.0429 (16)	0.0123 (17)	0.0101 (12)	0.0069 (16)
C6	0.0546 (17)	0.078 (2)	0.0499 (16)	0.0238 (15)	0.0055 (13)	0.0068 (15)
C7	0.0394 (14)	0.0433 (14)	0.0385 (12)	0.0043 (11)	0.0007 (11)	−0.0047 (10)
C8	0.0416 (16)	0.0557 (19)	0.0747 (19)	−0.0022 (12)	0.0023 (12)	0.0114 (15)
C9	0.0506 (16)	0.070 (2)	0.089 (2)	−0.0053 (15)	0.0082 (16)	0.0075 (17)
C10	0.0379 (16)	0.104 (3)	0.075 (2)	0.0028 (16)	−0.0055 (13)	−0.010 (2)
C11	0.0489 (16)	0.084 (2)	0.0561 (16)	0.0138 (17)	−0.0051 (12)	0.0061 (17)
C12	0.0495 (16)	0.0620 (18)	0.0552 (16)	0.0061 (14)	−0.0051 (11)	0.0098 (16)
C13	0.0404 (13)	0.0429 (15)	0.0437 (14)	0.0034 (12)	0.0023 (11)	−0.0019 (11)
C14	0.0426 (14)	0.0415 (15)	0.0396 (13)	0.0057 (12)	0.0020 (11)	−0.0025 (11)
C15	0.0599 (18)	0.062 (2)	0.0528 (16)	−0.0095 (15)	−0.0029 (13)	0.0043 (14)
C16	0.073 (2)	0.078 (2)	0.075 (2)	−0.0156 (18)	0.0113 (18)	0.022 (2)
C17	0.094 (2)	0.090 (3)	0.0482 (19)	0.011 (2)	0.0187 (16)	0.0196 (19)
C18	0.102 (2)	0.079 (2)	0.0459 (17)	0.015 (2)	0.0012 (18)	−0.0021 (16)
C19	0.0699 (19)	0.0531 (18)	0.0486 (16)	0.0012 (14)	0.0012 (13)	−0.0011 (14)
C20	0.093 (2)	0.066 (2)	0.0620 (18)	−0.0067 (18)	−0.0045 (16)	0.0123 (16)

### Geometric parameters (Å, °)

**Table:**

S1—O1	1.4291 (17)	C10—C11	1.495 (4)
S1—O2	1.4321 (17)	C10—H10A	0.9700
S1—N1	1.6219 (18)	C10—H10B	0.9700
S1—C1	1.758 (2)	C11—C12	1.520 (3)
N1—C13	1.467 (3)	C11—H11A	0.9700
N1—C7	1.483 (3)	C11—H11B	0.9700
C1—C6	1.371 (3)	C12—H12A	0.9700
C1—C2	1.372 (3)	C12—H12B	0.9700
C2—C3	1.374 (3)	C13—C14	1.507 (3)
C2—H2	0.9300	C13—H13A	0.9700
C3—C4	1.370 (3)	C13—H13B	0.9700
C3—H3	0.9300	C14—C19	1.372 (3)
C4—C5	1.374 (3)	C14—C15	1.381 (3)
C4—C20	1.502 (3)	C15—C16	1.382 (4)
C5—C6	1.379 (3)	C15—H15	0.9300
C5—H5	0.9300	C16—C17	1.367 (4)
C6—H6	0.9300	C16—H16	0.9300
C7—C8	1.513 (3)	C17—C18	1.364 (4)
C7—C12	1.515 (3)	C17—H17	0.9300
C7—H7	0.9800	C18—C19	1.377 (4)
C8—C9	1.528 (3)	C18—H18	0.9300
C8—H8A	0.9700	C19—H19	0.9300
C8—H8B	0.9700	C20—H20A	0.9600
C9—C10	1.514 (4)	C20—H20B	0.9600
C9—H9A	0.9700	C20—H20C	0.9600
C9—H9B	0.9700		
			
O1—S1—O2	119.55 (12)	C9—C10—H10A	109.5
O1—S1—N1	107.27 (10)	C11—C10—H10B	109.5
O2—S1—N1	107.05 (10)	C9—C10—H10B	109.5
O1—S1—C1	106.61 (10)	H10A—C10—H10B	108.0
O2—S1—C1	107.08 (11)	C10—C11—C12	111.3 (2)
N1—S1—C1	108.96 (10)	C10—C11—H11A	109.4
C13—N1—C7	119.09 (18)	C12—C11—H11A	109.4
C13—N1—S1	119.41 (15)	C10—C11—H11B	109.4
C7—N1—S1	118.11 (14)	C12—C11—H11B	109.4
C6—C1—C2	118.9 (2)	H11A—C11—H11B	108.0
C6—C1—S1	120.3 (2)	C7—C12—C11	110.9 (2)
C2—C1—S1	120.71 (18)	C7—C12—H12A	109.5
C1—C2—C3	120.1 (2)	C11—C12—H12A	109.5
C1—C2—H2	119.9	C7—C12—H12B	109.5
C3—C2—H2	119.9	C11—C12—H12B	109.5
C4—C3—C2	121.9 (2)	H12A—C12—H12B	108.1
C4—C3—H3	119.0	N1—C13—C14	114.46 (18)
C2—C3—H3	119.0	N1—C13—H13A	108.6
C3—C4—C5	117.2 (2)	C14—C13—H13A	108.6
C3—C4—C20	121.5 (3)	N1—C13—H13B	108.6
C5—C4—C20	121.3 (2)	C14—C13—H13B	108.6
C4—C5—C6	121.7 (2)	H13A—C13—H13B	107.6
C4—C5—H5	119.2	C19—C14—C15	118.4 (2)
C6—C5—H5	119.2	C19—C14—C13	120.5 (2)
C1—C6—C5	120.1 (3)	C15—C14—C13	121.1 (2)
C1—C6—H6	120.0	C14—C15—C16	120.2 (3)
C5—C6—H6	120.0	C14—C15—H15	119.9
N1—C7—C8	113.73 (19)	C16—C15—H15	119.9
N1—C7—C12	110.59 (18)	C17—C16—C15	121.0 (3)
C8—C7—C12	111.7 (2)	C17—C16—H16	119.5
N1—C7—H7	106.8	C15—C16—H16	119.5
C8—C7—H7	106.8	C18—C17—C16	118.8 (3)
C12—C7—H7	106.8	C18—C17—H17	120.6
C7—C8—C9	110.4 (2)	C16—C17—H17	120.6
C7—C8—H8A	109.6	C17—C18—C19	120.8 (3)
C9—C8—H8A	109.6	C17—C18—H18	119.6
C7—C8—H8B	109.6	C19—C18—H18	119.6
C9—C8—H8B	109.6	C14—C19—C18	120.9 (3)
H8A—C8—H8B	108.1	C14—C19—H19	119.6
C10—C9—C8	111.1 (2)	C18—C19—H19	119.6
C10—C9—H9A	109.4	C4—C20—H20A	109.5
C8—C9—H9A	109.4	C4—C20—H20B	109.5
C10—C9—H9B	109.4	H20A—C20—H20B	109.5
C8—C9—H9B	109.4	C4—C20—H20C	109.5
H9A—C9—H9B	108.0	H20A—C20—H20C	109.5
C11—C10—C9	110.9 (2)	H20B—C20—H20C	109.5
C11—C10—H10A	109.5		
			
O1—S1—N1—C13	159.58 (16)	S1—N1—C7—C8	−101.8 (2)
O2—S1—N1—C13	30.12 (19)	C13—N1—C7—C12	−69.2 (3)
C1—S1—N1—C13	−85.37 (18)	S1—N1—C7—C12	131.65 (18)
O1—S1—N1—C7	−41.37 (19)	N1—C7—C8—C9	178.9 (2)
O2—S1—N1—C7	−170.83 (17)	C12—C7—C8—C9	−55.1 (3)
C1—S1—N1—C7	73.68 (19)	C7—C8—C9—C10	55.6 (3)
O1—S1—C1—C6	16.6 (2)	C8—C9—C10—C11	−56.8 (3)
O2—S1—C1—C6	145.7 (2)	C9—C10—C11—C12	56.8 (3)
N1—S1—C1—C6	−98.9 (2)	N1—C7—C12—C11	−177.1 (2)
O1—S1—C1—C2	−162.71 (19)	C8—C7—C12—C11	55.2 (3)
O2—S1—C1—C2	−33.6 (2)	C10—C11—C12—C7	−56.0 (3)
N1—S1—C1—C2	81.8 (2)	C7—N1—C13—C14	91.6 (2)
C6—C1—C2—C3	−0.3 (4)	S1—N1—C13—C14	−109.5 (2)
S1—C1—C2—C3	179.01 (18)	N1—C13—C14—C19	−123.0 (2)
C1—C2—C3—C4	−1.0 (4)	N1—C13—C14—C15	58.5 (3)
C2—C3—C4—C5	1.6 (4)	C19—C14—C15—C16	−1.3 (4)
C2—C3—C4—C20	−176.6 (2)	C13—C14—C15—C16	177.2 (2)
C3—C4—C5—C6	−1.0 (4)	C14—C15—C16—C17	0.4 (5)
C20—C4—C5—C6	177.2 (3)	C15—C16—C17—C18	0.4 (5)
C2—C1—C6—C5	0.9 (4)	C16—C17—C18—C19	−0.4 (5)
S1—C1—C6—C5	−178.4 (2)	C15—C14—C19—C18	1.3 (4)
C4—C5—C6—C1	−0.2 (4)	C13—C14—C19—C18	−177.2 (3)
C13—N1—C7—C8	57.3 (3)	C17—C18—C19—C14	−0.5 (4)

### Hydrogen-bond geometry (Å, °)

**Table:**

*D*—H···*A*	*D*—H	H···*A*	*D*···*A*	*D*—H···*A*
C7—H7···O1	0.98	2.38	2.903 (3)	113
